# Wyburn-Mason Syndrome: A Narrative Review

**DOI:** 10.7759/cureus.68070

**Published:** 2024-08-28

**Authors:** Yasmin Shameem, Saleha Irshad, Nimrah Mirza, Natalie Hassan

**Affiliations:** 1 Paediatrics, Anglia Ruskin University, Chelmsford, GBR; 2 Critical Care, Basildon University Hospital, Basildon, GBR

**Keywords:** retinocephalic vascular malformation syndrome, arteriovenous malformation, racemose angiomatosis, bonnett-bechaume-blanc syndrome, wyburn-mason syndrome

## Abstract

Wyburn-Mason syndrome is a rare congenital disorder characterized by arteriovenous malformations (AVMs) in the retina, brain, and occasionally the skin. The syndrome results from embryonic vascular abnormalities and presents with a wide spectrum of clinical manifestations, classified into three groups based on severity. Diagnosis relies heavily on imaging techniques, with optical coherence tomography (OCT) and cerebral angiography playing crucial roles. Management is typically conservative due to the stability of most AVMs, but intervention may be necessary when the rupture risk of intracranial AVMs exceeds 2.2% per year. Treatment options include endovascular embolization, surgical resection, and emerging therapies like intravitreal injections. This review emphasizes the importance of a multidisciplinary approach involving ophthalmologists, neurologists, and interventional radiologists, as well as regular monitoring of asymptomatic AVMs to optimize patient outcomes and quality of life.

## Introduction and background

Wyburn-Mason syndrome (WMS), also known as Bonnet-Dechaume-Blanc syndrome, is a rare non-hereditary congenital neurocutaneous disorder characterised by vascular lesions known as arteriovenous malformations (AVMs) in the retina, brain, and/or skin [1]. Although the majority of AVMs reported in WMS are unilateral, bilateral occurrences have also been documented [2, 3]. The clinical presentation of WMS is highly variable, largely depending on the location and size of the AVMs, with symptoms ranging from visual impairment to neurological symptoms such as stroke and focal neurological deficits. Symptoms typically manifest in the second or third decade of life when AVMs become symptomatic, thus rendering the diagnosis challenging. At present, the exact incidence and prevalence of WMS in the general population remain unknown. This paper aims to investigate WMS through a narrative review of available literature, aiming to provide a comprehensive overview of the disorder's clinical features, diagnostic challenges, and current management strategies.

## Review

This narrative review encompasses 34 case reports sourced from published literature on PubMed from 1980 to 2023. Case reports that are included in this narrative review are patients with confirmed WMS. Other types of neurocutaneous syndromes associated with vascular malformations, such as Sturge-Weber, Klippel-Trenaunay-Weber syndromes, hereditary hemorrhagic telangiectasia, and blue rubber bleb naevus syndrome, were excluded. Case reports that did not identify the sex or the age upon diagnosis of the patients were also excluded. Data collated from the case reports included different parameters such as gender, age, presentation, comorbidities, associated syndromes, investigations, management, and complications (Table 1).

**Table 1:** Epidemiological, Biological Characteristics, and Management Plan of 38 Patients Diagnosed With Wyburn-Mason Syndrome. OCT: Optical Coherence Tomography; AVM: Arteriovenous Malformation; F: Female; M: Male; Y: Yes; N: No; RAPD: Relative Afferent Pupillary Defect

Author	Year	Age at Diagnosis of WMS	Sex	Presenting Complaint	Dilated Funduscopic	Fluorescein Angiography	MRI	Coronary Angiography	OCT	CT head	AVM location	Management
Augsburger et al. [4]	1980	26	F	Asymptomatic	Y	Y	N	N	N	Y	Unilateral Retinal & Unilateral Intracranial	Conservative
Kim et al. [5]	1998	6	M	Reduced Visual Acuity	Y	Y	Y	Y	N	N	Bilateral Retinal & Unilateral Intracranial	Not Reported
Sundaralingam and Muthukumar [6]	1998	12	M	Reduced Visual Acuity, Exophthalmos, Headaches, RAPD	Y	N	N	Y	N	Y	Unilateral Retinal & Unilateral Intracranial	Conservative
Chan et al. [7]	2004	9	F	Loss of Consciousness	Y	N	N	N	N	Y	Unilateral Retinal & Unilateral Intracranial	Not Reported
Reck et al. [8]	2005	7	F	Reduced Visual Acuity	Y	N	Y	Y	N	N	Unilateral Retinal & Unilateral Intracranial	Conservative
Lee et al. [9]	2007	12	F	Reduced Visual Acuity	Y	N	N	Y	N	N	Unilateral Retinal & Unilateral Intracranial	Conservative
Cortnum et al. [3]	2008	30	M	Headache	Y	N	N	Y	N	Y	Unilateral Retinal & Bilateral Intracranial	Not Reported
Matsuo et al. [10]	2008	31	M	Reduced Visual Acuity, Cheek Swelling, Exophthalmos	Y	N	Y	N	N	N	Unilateral Retinal	Catheter Embolization
Skorin and Simmons [11]	2008	22	M	Asymptomatic	Y	Y	Y	N	N	N	Unilateral Retinal	Conservative
Keswani et al. [12]	2009	9	M	Headache	Y	N	N	N	N	Y	Unilateral Retinal & Unilateral Intracranial	Conservative
Medina et al. [13]	2010	27	F	Reduced Visual Acuity	Y	Y	N	N	N	N	Unilateral Retinal	Posterior Vitrectomy with Retinal Reattachment
Rasalkar and Paunipagar [14]	2010	14	F	Reduced Visual Acuity, Headache	Y	N	N	Y	N	N	Unilateral Retinal & Unilateral Intracranial	Not Reported
Liu et al. [15]	2012	6	F	Reduced Visual Acuity, RAPD, Hemiparesis and Parasthesias	Y	Y	Y	Y	N	N	Unilateral Retinal & Unilateral Intracranial	Radiotherapy
Madey et al. [16]	2012	10	M	Monoplegia	N	N	Y	N	N	N	Unilateral Intracranial & Spinal	Not Reported
Tlucek et al. [17]	2012	12	F	Reduced Visual Acuity, RAPD	Y	N	Y	Y	Y	N	Unilateral Retinal	Conservative
Vucic et al. [18]	2012	11	M	Strabismus	Y	N	Y	N	N	N	Unilateral Retinal & Unilateral Intracranial	Conservative
Fileta et al. [19]	2014	12	F	Asymptomatic	Y	N	N	N	Y	N	Unilateral Retinal	Not Reported
Chowaniec et al. [20]	2015	5	F	Reduced Visual Acuity, RAPD	Y	N	Y	N	Y	N	Unilateral Retinal & Unilateral Intracranial	Conservative
Chowaniec et al. [20]	2015	10	M	Asymptomatic	Y	N	Y	N	Y	N	Unilateral Retinal	Conservative
Iwata et al. [21]	2015	11	F	Reduced Visual Acuity	Y	Y	Y	Y	Y	N	Unilateral Retinal & Unilateral Intracranial	Not Reported
Onder et al. [22]	2015	14	M	Reduced Visual Acuity	Y	Y	Y	N	Y	N	Unilateral Retinal	Medical Management
Bhojwani et al. [23]	2016	18	M	Reduced Visual Acuity, Headache	Y	Y	Y	N	Y	N	Unilateral Retinal & Unilateral Intracranial	Not Reported
Kolomeyer et al. [24]	2016	6	M	Reduced Visual Acuity, RAPD	Y	N	Y	N	Y	N	Unilateral Retinal & Unilateral Intracranial	Not Reported
Weng et al. [25]	2017	10	M	Headache	Y	N	Y	N	N	N	Unilateral Retinal & Unilateral Intracranial	Not Reported
Pangtey et al. [2]	2018	37	M	Reduced Visual Acuity	Y	Y	Y	N	Y	N	Bilateral Retinal	Intravitreal bevacizumab
Naik et al. [26]	2019	21	F	Reduced Visual Acuity	Y	N	N	N	Y	N	Unilateral Retinal	Intravitreal ranibizumab
Battista et al. [27]	2020	16	F	Reduced Visual Acuity	Y	Y	N	N	Y	N	Unilateral Retinal	Not Reported
Fortes et al. [28]	2020	11	M	Reduced Visual Acuity, RAPD	Y	Y	Y	N	Y	N	Unilateral Retinal	Intravitreal bevacizumab
Yamao and Kusaka [29]	2020	11	F	Reduced Visual Acuity	Y	N	Y	N	Y	N	Unilateral Retinal	Not Reported
Ouedrago et al. [30]	2020	5	M	Headaches with Prolonged Seizures	Y	N	Y	N	N	Y	Unilateral Intracranial	Surgical Management
Ouedrago et al. [30]	2020	36	M	Headaches with Exophthalmos	Y	N	N	Y	N	N	Unilateral Intracranial	Catheter Embolization
Ouedrago et al. [30]	2020	35	M	Reduced Visual Acuity, Headache	N	N	Y	Y	N	N	Unilateral Intracranial	Radiotherapy
Choo et al. [31]	2022	5	F	Strabismus	Y	Y	Y	Y	Y	N	Unilateral Retinal & Unilateral Intracranial	Conservative
Vaithialingam et al. [1]	2023	24	F	Exophthalmos	Y	N	N	Y	N	N	Unilateral Retinal & Unilateral Intracranial	Catheter Emoblization

The age at which patients were diagnosed with WMS varied widely, spanning from five to 27 years, with a mean age of 15.6 years and a standard deviation of 9.53. The review revealed a relatively balanced gender distribution, with 47% (16 cases) of females compared to 53% (18 cases) of males. The mean age of female patients was 13.6 years, whereas the mean age of male patients was 17.4 years.

The clinical presentation of the cases varied, with the majority (59%; 20 cases) presenting with reduced visual acuity, making it the most frequently observed symptom. This was the sole presenting complaint in 50% (10 cases). Four cases (20%) had reduced visual acuity associated with a relative afferent pupillary defect (RAPD); three cases (15%) had associated headaches; one case (5%) had associated cheek swelling and exophthalmos; one case (5%) had a combination of reduced visual acuity and exophthalmos, intermittent headaches along with RAPD, and one case (5%) had a reduced visual acuity associated with RAPD, hemiparesis, and parathesias. Five cases (14%) presented with headaches, one of which was associated with exophthalmos, one with prolonged seizures, and one with hemiplegia. Two cases (6%) presented with strabismus; one case (3%) presented with loss of consciousness; one case (3%) presented with monoplegia; one case (3%) presented with exophthalmos alone; and four cases (12%) were asymptomatic, and the diagnosis was made after incidental findings.

**Figure 1:**
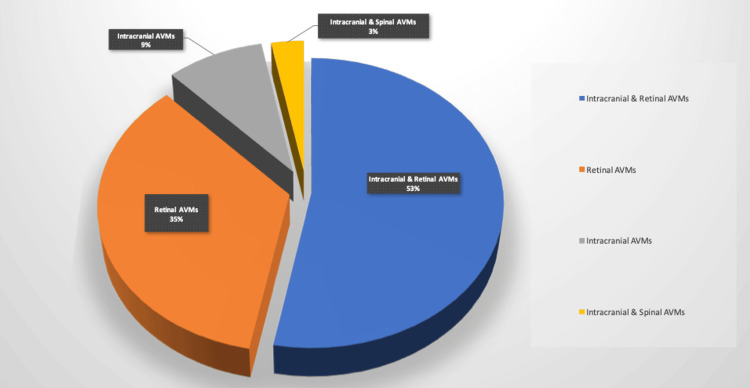
Location of AVMSs in 38 Patients Diagnosed With Wyburn-Mason Syndrome AVM: Arteriovenous Malformation This Image Has Been Created by the Authors.

Eighteen cases (53%) were reported to have both retinal and intracranial AVMs; of those, the majority (89%; N = 16) were unilateral, with one case having unilateral intracranial AVMs with bilateral retinal AVMs and one with bilateral intracranial AVMs with unilateral retinal AVMs. Twelve cases (35%) had isolated retinal AVMs, with the majority (92%, N = 11) being unilateral and only one being bilateral. Three cases (9%) had isolated unilateral intracranial AVMs, and one case (3%) had unilateral intracranial and spinal AVMs (Figure 1).

Different investigations were used to aid with the diagnosis of WMS. Among the study group, 94% of patients (N = 32) had a dilated funduscopy, 35% of patients (N = 12) had a fluorescein angiography, and 62% of patients (N = 21) had an MRI. Cerebral angiography was used in 38% of patients (N = 13). Optical coherence tomography (OCT) was used in 41% of cases (N = 14), and a head CT scan was used in 18% of cases (N = 6).

**Figure 2:**
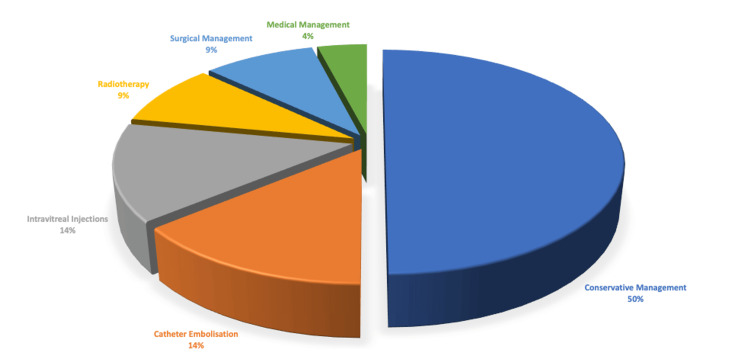
Management Modalities of 38 patients with Wyburn-Mason Syndrome This Image Has Been Created by the Authors.

The management modalities have differed across the case reports; these were discussed in 22 of the 34 cases. Fifty per cent of these cases (N = 11) were managed conservatively through observation with routine ophthalmic follow-up; 14% (N = 3) received catheter embolisation; 14% (N = 3) received intravitreal injections; 9% of the cases (N = 2) received radiotherapy; 9% (N = 2) had surgeries; and one case (4%) received medical management with eye drops (Figure 2).

Wyburn-Mason syndrome is caused by sporadic abnormalities involving the primitive vascular mesoderm, developing optic cup, and anterior neural tube prior to the seventh week of embryogenesis [1]. As a result, retinal and intracranial AVMs tend to be ipsilateral, with the midbrain most commonly affected [28]. Subsequently, the clinical presentation and severity of WMS, which is characterised by AVMs, have a very wide spectrum. To guide the clinical approach for patients with AVMs, they have been classified into three groups which are as follows [32]: Group 1 is defined by an abnormal capillary plexus between the artery and the vein. These lesions tend to be small, with patients asymptomatic and intracranial involvement uncommon. Group 2 is defined by arteriovenous malformations without an intervening capillary bed between the artery and the vein [32]. This group is associated with an increased risk of retinal complications such as retinal oedema, haemorrhage, and vision loss [32]. The risk of intracranial involvement in this group is low [32]. Group 3 is defined as extensive AVMs with dilated and tortuous vessels with no distinction between the artery and the vein [32]. This group has a high risk of vision loss due to retinal decompensation or retinal compression of the nerve fibre layer, optic nerve, or other vessels. It also has a high risk of intracranial AVMs which are most often found in the midbrain [32]. The presence of Group 3 AVMs is used to make a formal diagnosis of WMS and is often associated with significant morbidity.

Why are certain investigations used?

Imaging plays an important role in the assessment of AVMs and serves to inform the best-suited approach to patient management. Most retinal AVMs in WMS are typically picked up during bedside ophthalmoscopy, although fluorescein angiography may be required to demonstrate the presence of smaller lesions [33]. However, those investigations are of limited use in evaluating the AVMs and determining their progression. For this purpose, OCT is recommended as it allows the visualisation of the inner retinal and photoreceptor layers, which is useful in long-term patient follow-up [20]. Furthermore, it can detect associated complications of retinal AVMs such as macular oedema and serous retinal detachment [33]. In addition to that, there has been one patient with WMS reported to have an increase in the choroidal thickness, which was detected using OCT, although more studies are needed to determine whether a thickened choroid is a characteristic of WMS [21]. 

Most retinal AVMs remain stable, with a small risk of haemorrhage that can affect the vision, and as such, ophthalmologists can primarily diagnose WMS; however, a thorough neurological workup to exclude the presence of intracranial AVMs must be carried out [20, 28]. The intracranial AVMs in cases of WMS are found to be more extensive and located deeper in the brain in comparison to the average isolated intracranial AVM [19]. It is recommended that all asymptomatic intracranial AVMs in WMS be managed as isolated asymptomatic intracranial AVMs, which have an estimated rupture rate of 2.2% per year [8]. It is important to note that changes in the retinal AVMs as an indication of concurrent changes in intracranial AVMs in patients with WMS are yet to be determined [4].

Intracranial AVMs can be detected using different imaging modalities, including CT, MRI, magnetic resonance angiography (MRA), and cerebral angiography. Among those, cerebral angiography remains the gold standard investigation of choice for intracranial AVMs as it can delineate the size, location, and characteristics of the feeding arteries and draining veins and thus is useful for understanding both the angioarchitecture and the haemodynamics of the AVM [34]. However, due to the risks of cerebral angiography, this investigation is typically reserved for symptomatic patients, whereby it is used in surgical planning [28]. 

Intracranial AVMs, although congenital, typically become symptomatic in the second or third decade [28]. In patients who present with focal neurological deficits, a CT scan is usually the first imaging modality used, particularly sensitive in the detection of haemorrhage. Subarachnoid haemorrhage from a cerebral AVM rupture is the most feared complication and may be associated with significant morbidity [3].

In 2012, WMS was reported in a patient with a background of Duane syndrome, and as such, it was recommended that any patient presenting with strabismus always be examined for retinal and optic nerve disorders, and if retinal AVMs are detected, neurological examination and imaging are mandatory [18].

Why observe instead of operating?

The presence of both a retinal and an intracranial AVM merits the diagnosis of WMS, and the treatment is often based on the location of the AVMs and the corresponding clinical symptoms [33].

Conservative management remains the mainstay approach to patient management after a diagnosis of WMS has been confirmed. The choice of observation is often indicated for both symptomatic and asymptomatic patients due to the stability of the AVMs. A follow-up observation of 27 years in one case reported no changes in the retinal or cerebral AVMs [35]. On the other hand, another report that documented the long-term follow-up of three patients with WMS described a severe deterioration in the neurological functioning of all three patients [30].

Based on location, intracranial AVMs can cause vision loss secondary to strokes or disruption of the anterior visual pathway because these AVMs tend to be located in the distribution of the optic tracts and optic radiations [31]. The rupture of these AVMs may lead to spontaneous thrombosis and haemorrhage, which require prompt treatment [3]. 

In contrast, retinal AVMs are often stable with a smaller risk of haemorrhage, and therefore surveillance is preferred to active management [22]. However, retinal AVMs can exhibit increased tortuosity that can give rise to complications that require active medical intervention [22].

Active management of AVMs in patients diagnosed with WMS includes radiation therapy, embolisation, surgical resection, or a combination of those and is necessitated if the rupture rate of the AVM exceeds 2.2% per year [30].

In 2012, the use of CyberKnife treatment (30 Gy) to a right orbital AVM located distal to the right ophthalmic artery was reported; however, the treatment was unsuccessful and the visual acuity of the patient severely deteriorated afterwards [15]. As a result, it is suggested that surgical resection is best for AVMs less than 3 cm, with complications occurring more frequently with AVMs that are greater than 6 cm [15].

Successful posterior vitrectomy was reported in a patient with WMS who presented with moderate vitreous haemorrhage and retinal detachment with a superior tear; this was the first case to describe rhegmatogenous retinal detachment in a patient with WMS [13]. The bleeding risk during vitrectomy was mitigated with low aspiration rates and careful detachment of the posterior hyaloid [13].

Another approach that was reported is intravitreal injection of bevacizumab, which helps improve the intraretinal fluid and neurosensory detachment, leading to vision improvement [2]. Another case reported the successful use of intravitreal ranibizumab [26]. On the other hand, a posterior subtenon triamcinolone injection was documented to reduce the cystoid macular oedema and successfully restore vision in a 14-year-old patient with WMS [22].

In summary, patients with orbital AVMs who present with forehead swelling and proptosis may seek surgical intervention for cosmetic reasons; however, catheter embolisation remains the most favoured treatment of choice for AVMs where an intervention is required [1].

What is the best approach for patients with WMS?

The best approach for patients with WMS is multifaceted and requires multidisciplinary input. The detection of retinal AVMs requires urgent ophthalmology involvement and warrants a mandatory neurological workup that facilitates examination and imaging of the orbits, paranasal sinuses, and brain to exclude cerebral AVMs [18]. Sudden rupture of intracranial AVMs can be fatal and may necessitate an emergency craniotomy, which has poor prognostic outcomes [33].

It is recommended that asymptomatic intracranial AVMs are periodically followed up with an intervention favoured only if the rupture rate exceeds 2.2% per year [1]. For patients where an intervention is required, endovascular catheter embolisation is preferable as it is less invasive and has fewer intraoperative complications [1, 33]. 

Traditional methods of treatment, such as radiotherapy, have been used in WMS patients; however, more research is needed to determine its therapeutic benefit. Radiotherapy can have severe endocrine effects as it can disrupt the hypothalamus-pituitary axis, which makes it an undesirable choice for paediatric patients [36].

Ultrawide field colour imaging has emerged as a novel imaging modality whereby imaging is conducted via a non-contrast ophthalmoscope, which is advantageous in paediatric patients as it negates the need for sedation and has been used in patients as young as three years of age [2].

## Conclusions

In summary, WMS is a rare congenital disorder with poor prognostic outcomes when associated with spontaneous haemorrhage due to the sudden rupture of retinal and intracranial AVMs. As such, a multidisciplinary approach is essential for effective management. It is important that asymptomatic AVMs be monitored regularly with surgical intervention warranted for those who are symptomatic to mitigate risks and improve patient outcomes. By coordinating care among specialists and tailoring treatment plans to individual needs, the management of WMS can be optimised, enhancing the quality of life and prognosis for affected individuals.
